# A Dual Role for KRT81: A miR-SNP Associated with Recurrence in Non-Small-Cell Lung Cancer and a Novel Marker of Squamous Cell Lung Carcinoma

**DOI:** 10.1371/journal.pone.0022509

**Published:** 2011-07-25

**Authors:** Marc Campayo, Alfons Navarro, Nuria Viñolas, Rut Tejero, Carmen Muñoz, Tania Diaz, Ramon Marrades, Maria L. Cabanas, Josep M. Gimferrer, Pere Gascon, Jose Ramirez, Mariano Monzo

**Affiliations:** 1 Department of Medical Oncology, Institut Clinic Malalties Hemato-Oncològiques (ICMHO), Hospital Clinic de Barcelona, University of Barcelona, Institut d'Investigacions Biomèdiques August Pi i Sunyer (IDIBAPS), Barcelona, Spain; 2 Human Anatomy and Embryology Unit, Laboratory of Molecular Oncology and Embryology, School of Medicine, University of Barcelona, Institut d'Investigacions Biomèdiques August Pi i Sunyer (IDIBAPS), Barcelona, Spain; 3 Department of Pneumology, Institut Clínic del Tórax (ICT), Hospital Clinic de Barcelona, University of Barcelona, Institut d'Investigacions Biomèdiques August Pi i Sunyer (IDIBAPS), CIBER de Enfermedades Respiratorias (CIBERES), Barcelona, Spain; 4 Department of Pathology, Centro de Diagnóstico Biomédico (CDB), Hospital Clinic de Barcelona, University of Barcelona, Institut d'Investigacions Biomèdiques August Pi i Sunyer (IDIBAPS), CIBER de Enfermedades Respiratorias (CIBERES), Barcelona, Spain; 5 Department of Thoracic Surgery, Institut Clínic del Tórax (ICT), Hospital Clinic de Barcelona, University of Barcelona, Barcelona, Spain; Virginia Commonwealth University, United States of America

## Abstract

MicroRNAs (miRNAs) play an important role in carcinogenesis through the regulation of their target genes. miRNA-related single nucleotide polymorphisms (miR-SNPs) can affect miRNA biogenesis and target sites and can alter microRNA expression and functions. We examined 11 miR-SNPs, including 5 in microRNA genes, 3 in microRNA binding sites and 3 in microRNA-processing machinery components, and evaluated time to recurrence (TTR) according to miR-SNP genotypes in 175 surgically resected non-small-cell lung cancer (NSCLC) patients. Significant differences in TTR were found according to *KRT81* rs3660 (median TTR: 20.3 months for the CC genotype versus 86.8 months for the CG or GG genotype; P = 0.003) and *XPO5* rs11077 (median TTR: 24.7 months for the AA genotype versus 73.1 months for the AC or CC genotypes; P = 0.029). Moreover, when patients were divided according to stage, these differences were maintained for stage I patients (P = 0.002 for *KRT81* rs3660; P<0.001 for *XPO5* rs11077). When patients were divided into sub-groups according to histology, the effect of the *KRT81* rs3660 genotype on TTR was significant in patients with squamous cell carcinoma (P = 0.004) but not in those with adenocarcinoma. In the multivariate analyses, the *KRT81* rs3660 CC genotype (OR = 1.8; P = 0.023) and the *XPO5* rs11077 AA genotype (OR = 1.77; P = 0.026) emerged as independent variables influencing TTR. Immunohistochemical analyses in 80 lung specimens showed that 95% of squamous cell carcinomas were positive for KRT81, compared to only 19% of adenocarcinomas (P<0.0001). In conclusion, miR-SNPs are a novel class of SNPs that can add useful prognostic information on the clinical outcome of resected NSCLC patients and may be a potential key tool for selecting high-risk stage I patients. Moreover, KRT81 has emerged as a promising immunohistochemical marker for the identification of squamous cell lung carcinoma.

## Introduction

Lung cancer is the first cause of cancer death worldwide[1]. About 85% of patients have non-small-cell lung cancer (NSCLC) and less than 30% are diagnosed with early-stage disease. The main treatment for early-stage disease is surgery, but even when a complete surgical resection is possible, 20–75% of NSCLC patients will relapse [2]. Given this high rate of relapse, biomarkers to predict the risk of disease progression are needed, especially in stage I, where adjuvant chemotherapy is not routinely administered but where it may be effective in certain subgroups of patients [3].

MicroRNAs (miRNAs) are short non-coding RNAs that regulate post-transcriptional gene expression by binding primarily to the 3′untranslated region (UTR) of their target mRNA and repressing its translation. Several proteins are active in the biogenesis of miRNAs. Briefly, miRNAs are translated by an RNA polymerase II to long primary transcripts (pri-miRNA) and processed in the nucleus by the RNase III Drosha in pre-miRNAs (70–100 nucleotides); the pre-miRNA is transported to the cytoplasm by the XPO5, where the RNase III Dicer generates a duplex molecule of 21-25 nucleotides in length. Through the association with the complex RNA-induced silencing complex (RISC), one of these 2 chains (the mature miRNA) will guide RISC to the target mRNA [4], [5]. miRNAs play important roles in the regulation of such crucial processes as development, cell proliferation, differentiation and apoptosis. Growing evidence shows that miRNAs are aberrantly expressed in human cancers, including NSCLC [6], [7], [8], [9], [10], [11], [12], [13], and they have been linked with the etiology and prognosis of many tumors [14]. Depending on their target genes, miRNAs can act either as oncogenes or tumor suppressor genes [15].

Various mechanisms can explain the deregulation of miRNAs observed in cancer, including genomic changes (deletions, amplifications, translocations), epigenetic changes, mutations/polymorphisms, transcriptional deregulation, and alterations in the miRNA biogenesis machinery [14], [16]. Single nucleotide polymorphisms (SNPs) that can affect miRNA functions, known as miR-SNPs, are found in miRNA genes, in miRNA binding sites (in 3′ UTR of the target gene) or in the components of the miRNA biogenesis machinery [17]. miR-SNPs can affect miRNA expression levels in different ways, resulting in loss or gain of miRNA function [18]. SNPs in miRNA genes can affect the pri-miRNA, pre-miRNA or mature miRNA sequence and can potentially modulate miRNA processing, alter mature miRNA levels or change miRNA-mRNA interactions [19], [20]; SNPs affecting the expression of proteins involved in miRNA biogenesis may alter the miRNAome in the cell [21]; and finally, SNPs in miRNA target sites, which are more frequent and more specific in the human genome, can disrupt or alter the miRNA-mediated repression of a target gene [22].

This novel class of SNPs opens up a new area of research in cancer biology and clinical oncology, especially in the study of disease progression, patient prognosis and treatment efficacy. Recently, various studies have shown that SNPs in miRNA networks can affect both the risk of developing various cancers [20] and also the prognosis of many tumors [23], [24], [25], [26].

In the present study, we have evaluated 11 SNPs (five in miRNA genes, three in miRNA binding sites, and three in miRNA-processing genes) in 175 surgically resected NSCLC patients and correlated our findings with time to recurrence (TTR) and overall survival (OS). In addition, in order to examine potential differences in expression according to histology, we examined the immunostaining pattern of KRT81 in 77 lung cancer specimens and three normal lung controls.

## Materials and Methods

### Study population and Ethics Statement

Between March 1996 and December 2009, 175 NSCLC patients underwent complete surgical resection in our institution. All patients had pathologically confirmed stage I-III disease. Approval for the study was obtained from the Institutional Review Board of the Hospital Clinic, Barcelona, Spain. Written informed consent was obtained from each participant in accordance with the Declaration of Helsinki.

### Selection of the miR-SNPs

We selected 11 SNPs in genes involved in miRNA regulatory pathways: SNPs in miRNA genes; SNPs in miRNA binding sites; and SNPs in miRNA-processing genes. Ten of the SNPs were selected according to the following requirements: firstly, a determined allele frequency for the European population and availability in the National Center for Biotechnology Information (NCBI) SNP database; secondly, a minor genotype frequency for the European population ≥ 0.05; and finally, either a known association with a differential susceptibility to cancer development or a differential expression in solid tumors. For the SNPs in miRNA binding sites, we selected three SNPs with an aberrant allelic frequency in human tumors. In addition, one SNP – in *MIR194-2* – was specifically chosen because it had been shown to be differentially expressed in lung cancer [11]. Table 1 summarizes the rationale for the SNP selection.

**Table 1:** Rationale for the selection of the miR-SNPs analyzed.

Location	Gene	rs NCBI(AB assay ID)	Rationale *
**miRNA genes**	*MIR194-2*	rs11231898(C__32062040_10)	miR-194 is differentially expressed in lung cancer [11] *
	*MIR196A2*	rs11614913(C__31185852_10)	Risk of head and neck, breast, lung and gastric cancers [25], [27], [44], [45]Poor survival in lung cancer [23]
	*MIR149*	rs2292832(C__11533078_1_)	miR-149 is differentially expressed in prostate cancer [46]
	*MIR423*	rs6505162(C__11613678_10)	Risk of bladder cancer [47]Decreased risk esophageal cancer [48]
	*MIR146A*	rs2910164(C__15946974_10)	Risk of papillary thyroid carcinoma [49]Risk of hepatocarcinoma [50]Risk of prostate cancer [51]
**miRNA binding sites**	*KRT81*	rs3660(C__11917951_20)	miRNA-binding SNPs with an aberrant SNP allele frequency in human cancers [36]
	*FAM179B*	rs1053667(C__11606996_1_)	miRNA-binding SNPs with an aberrant SNP allele frequency in human cancers [36]
	*AFF1*	rs17703261(C__32818766_10)	miRNA-binding SNPs with an aberrant SNP allele frequency in human cancers [36]
**miRNA-processing machinery**	*XPO5*	rs11077(C___3109165_1_)	Risk of esophageal cancer [48]
	*RAN*	rs14035(C__11351340_10)	Risk of esophageal cancer [48]
	*TRBP*	rs784567(C___9576934_20)	Risk of bladder cancer [47]

*One of the selection criteria was a described association with a differential susceptibility to cancer development. In MIR194-2, the association was with the miRNA containing the SNP, while in all other cases, the association was with the miR-SNP itself.

### DNA isolation, primers, probes and SNP analysis

DNA was obtained from paraffin-embedded tumor tissue using the commercial DNeasy tissue kit (Qiagen, Valencia, CA) following the manufacturer's protocol. To measure DNA quantity, a NanoDrop ND-1000 spectrophotometer (Thermo Fisher Scientific Inc., Waltham, MA) was used. Primers and probes were commercially available (TaqMan SNP Genotyping Assays, Applied Biosystems, Foster City, CA). SNP analysis was performed by allelic discrimination in an ABI PRISM 7500 Sequence detection system (Applied Biosystems).

### Immunohistochemistry

Immunohistochemistry was performed on formalin-fixed, paraffin-embedded tissue sections of 77 lung carcinomas and 3 normal lung controls from the Pathology Service of the Hospital Clinic of Barcelona after review by a thoracic pathologist . Five-µm-thick transverse sections of formalin-fixed, paraffin-embedded tissue were serially cut and mounted onto Dako Silanized Slides (S·3003; Dako, Glostrup, Denmark). For antigen retrieval, the sections were manually immersed in Target Retrieval solution, high pH (Dako) and heated in a water bath at 95–99°C for 20 min. Endogenous peroxidase activity was quenched by immersion in Dako Real Peroxidase-Blocking solution for 10 min. The tissue sections were incubated with primary antibody against KRT81 (dilution 1∶50; clone sc-100929; Santa Cruz Biotechnology, Santa Cruz, CA) for 30 min at room temperature. Immunoperoxidase staining was performed using Advance system/HRP (Dako) and Liquid DAB+ (Dako). Finally, sections were stained with hematoxylin. All slides were blindly scored by the same two pathologists using a 3-point system. The scoring system was as follows: 0, <5% of tumor cells staining; 1, 5% to 50% of tumor cells staining; 2, >50% of tumor cells staining. Uninterpretable results were eliminated from further consideration. Cases scored 1 or 2 were considered positive, and cases scored 0 were considered negative. Only cytoplasmic positivity was evaluated. Tissue sections from normal lung were used as positive controls while negative controls were obtained by incubating the sections without the primary antibody.

### Statistical analysis

The primary objective of the study was TTR. The secondary objective was OS. All statistical analyses were performed using PAS W Statistics 18 (SPSS Inc., Chicago, IL). TTR was calculated from the time of surgical treatment to the date of relapse or last follow-up. OS was calculated from the time of surgical treatment to the date of death or last follow-up. After surgery, patients without tumor progression were followed every 3 months for 2 years, then every 6 months until 5 years after surgery, and then annually. The log-rank test and Kaplan-Meier plots were used to evaluate the association of TTR and OS with each of the SNPs and clinical variables. A Cox multivariate analysis (enter method) was used to calculate the independent odds ratios for TTR and OS. In the immunohistochemical analyses, frequencies were compared by the Fisher's exact test. The level of significance was set at ≤0.05.

## Results

### Patient Characteristics

The analysis included 175 patients, 154 (88%) of whom were male. Median age was 65 years (range, 35–85). Twenty-four (13.7%) patients had Eastern Cooperative Oncology Group (ECOG) performance status (PS) 0 and 149 (85.2%) patients had PS 1. Ninety-eight (56%) patients had stage I disease. Eighty (45.7%) patients had adenocarcinoma and 84 (48%) had squamous cell carcinoma. One hundred and fifty-eight (90.3%) patients were active or former smokers. One hundred and thirty-two (75.4%) patients underwent a lobectomy or bilobectomy. Nine (5.1%) patients had received preoperative chemotherapy or chemoradiotherapy for resectable stage IIIA disease. Sixteen patients (9.1%) received adjuvant chemotherapy (13 for stage II or III disease and 3 for stage I disease with T>4 cm). Mean follow-up was 35 months (range, 2-160). After a follow-up of 160 months, disease recurrence had occurred in 75 (42.9%) patients (Table 2).

**Table 2:** Patient characteristics.

Characteristic	Value	N = 175N (%)	TTRP-value	OSP-value
**Sex**	Male	154 (88)	0.081	0.173
	Female	21 (12)		
**Age**	≤65	84 (48)	0.926	0.014
	>65	91 (52)		
**Performance Status**	0	24 (13.7)	0.989	0.360
	1	149 (85.2)		
	2	2 (1.1		
**Stage**	I	98 (56)	0.008	0.349
	II	40 (22.9)		
	III	37 (21.1)		
**Histology**	Adenocarcinoma	80 (45.7)	0.996	0.756
	Squamous cell carcinoma	84 (48)		
	Others	11 (6.3)		
**Smoking History**	Current smoker	72 (41.1)	0.648	0.828
	Former smoker	86 (49.2)		
	Never smoker	9 (5.1)		
	Unknown	8 (4.6)		
**Type of surgery**	Lobectomy/Bilobectomy	132 (75.4)	0.116	0.307
	Pneumonectomy	34 (19.4)		
	Atypical resection	9 (5.2)		
**Treatment**	Neoadjuvant*	9 (5.1)	0.492	0.339
	Adjuvant**	16 (9.1%)	0.997	0.716
**Recurrence**	No	100 (57.1)		
	Yes	75 (42.9)		

*chemotherapy or chemoradiotherapy; **chemotherapy.

### TTR, OS and miR-SNPs

**Figure 1:**
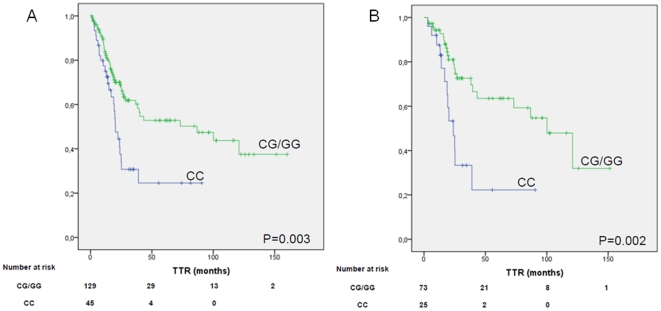
TTR according to *KRT81* rs3660 genotype (CC = wild-type; CG/GG = non wild-type). 1A: in all patients analyzed. IB: patients with stage I disease.

**Figure 2:**
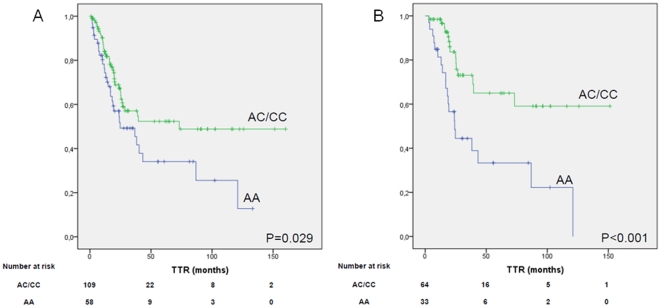
TTR according to *XPO5* rs11077 genotype (AA = wild-type; AC/CC = non wild-type). 1A: in all patients analyzed. IB: in patients with stage I disease.

Overall median TTR was 39.03 months (95% CI, 3.9–74.1), and median OS was 90.6 months (95% CI, 47.4–133.7). In univariate analyses including only clinical characteristics, stage was associated with TTR (P = 0.008) and age was associated with OS (P = 0.014). A non-significant trend towards an association between sex and TTR was also observed (P = 0.081). Table 3 shows the genotypic frequencies for all 11 miR-SNPs analyzed, both in the present study and as reported in NCBI SNP database (dbSNP) for the European population. Significant differences in TTR were found according to *KRT81* rs3660 genotype (P = 0.008; Figure S1). Given the similar distribution in TTR for patients with the CG and GG genotypes, these two groups were combined for further analyses. Median TTR for 45 patients (25.9%) with the CC genotype was 20.3 months versus 86.8 months for patients with the CG or GG genotype (P = 0.003; Figure 1A). Among 98 patients with stage I disease, median TTR was 23.9 months for 25 patients (25.5%) with the CC genotype versus 100.2 months for patients with the CG or GG genotype (P = 0.002; Figure 1B). We also observed a non-significant trend towards a differential TTR according to the *XPO5* rs11077 genotype (P = 0.077; Figure S2). Given the similar distribution in TTR for patients with the AC and CC genotypes, these two groups were combined for further analyses. A significantly shorter TTR was observed in patients with the *XPO5* rs11077 AA genotype; median TTR was 24.7 months for patients with the AA genotype, versus 73.1 months for those with the AC or CC genotype (P = 0.029; Figure 2A). Among 97 patients with stage I disease, median TTR was 24.13 months for 33 patients (34%) with the AA genotype but was not reached for those with the AC or CC genotype (P<0.001; Figure 2B). No other differences in TTR were observed according to any of the other genotypes analyzed. No significant differences in TTR were observed in stage II-III patients according to any of the SNPs analyzed.

**Table 3:** Genotypic frequencies in the present study and for the European Population in NCBI dbSNP.

Gene	Genotype	EP	N (%)	TTR P-value	OS P-value
***MIR194-2***	GG	100	161 (100)		
**rs11231898**	AG	-	-	-	-
**n = 161** *	AA	-	-		
***MIR196A2***	CC	31	66(38.1)		
**rs11614913**	CT	50	87(50.3)	0.798	0.227
**n = 173**	TT	19	20(11.6)		
***MIR149***	CC	56.7	93 (56)		
**rs2292832**	CT	35	50 (30.1)	0.502	0.845
**n = 166**	TT	8.3	23 (13.9)		
***MIR423***	AA	26.7	49 (28.8)		
**rs6505162**	AC	61.7	79 (46.5)	0.754	0.043
**n = 170**	CC	11.7	42 (24.7)		
***MIR146A***	GG	59.3	92 (52.9)		
**rs2910164**	CG	33.9	75 (43.1)	0.845	0.815
**n = 174**	CC	6.8	7 (4)		
***KRT81***	CC	36.7	45 (25.9)		
**rs3660**	CG	45	79 (45.4)	0.008	0.471
**n = 174**	GG	18.3	50 (28.7)		
***FAM179B***	TT	93.3	158 (90.8)		
**rs1053667**	CT	5	16 (9.2)	0.977	0.407
**n = 174**	CC	1.7	-		
***AFF1***	AA	59.1	-		
**rs17703261**	AT	22.7	133 (100)	-	-
**n = 133** *	TT	18.2	-		
***XPO5***	AA	41.7	58 (34.7)		
**rs11077**	AC	36.7	74 (44.3)	0.077	0.363
**n = 167**	CC	21.7	35 (21)		
***RAN***	CC	55	72 (49.3)		
**rs14035**	CT	36.7	65 (44.5)	0.263	0.202
**n = 146**	TT	8.3	9 (6.2)		
***TRBP***	CC	28.3	45 (26.2)		
**rs784567**	CT	46.7	91 (52.9)	0.985	0.636
**n = 172**	TT	25	36 (20.9)		

EP: frequencies (%) for European Population in NCBI dbSNP.

In some cases the genotype could not be determined; “n” indicates the number of patients genotyped in each case.

*In these two cases we discontinued the analysis since all patients analyzed had the same genotype.

Median OS was not reached for 49 patients with the *MIR423* rs6505162 AA genotype, compared to 61.6 months for 42 patients with the CC genotype and 90.5 months for those with the AC genotype (P = 0.043; Figure S3). No other differences in OS were observed according to any of the other genotypes analyzed.

### Multivariate analyses

All variables with a univariate TTR log-rank P≤0.1 (sex, stage, type of surgery, *KRT81* rs3660 genotype and *XPO5* rs11077 genotype) were included in the Cox multivariate analysis for TTR. Male sex (odds ratio [OR], 3.73; 95% CI, 1.4–9.9; P = 0.008), stage I disease (OR, 0.34; 95% CI, 0.18–0.65; P = 0.001), *KRT81* rs3660 CC genotype (OR,1.8; 95% CI, 1.08–2.99; P = 0.023) and *XPO5* rs11077 AA genotype (OR, 1.77; 95% CI,1.07–2.91; P = 0.026) emerged as independent variables for TTR (Table 4). All variables with a univariate OS log-rank P≤0.1 (sex, age and *MIR423* rs6505162 genotype) and disease stage were included in the Cox multivariate analysis for OS. Age ≤65 (OR, 0.48; 95% CI, 0.25–0.95; P = 0.036) and stage I disease (OR = 0.31, 95% CI 0.14–0.67; P = 0.003) were independent variables for OS.

**Table 4:** Multivariate analysis for TTR.

Variable	OR (95% CI)	P-value
Male sex	3.73 (1.4–9.9)	0.008
Stage I	0.34 (0.18–0.65)	0.001
*KRT81* rs3660 CC	1.8 (1.08–2.99)	0.023
*XPO5* rs11077 AA	1.77 (1.07–2.91)	0.026

### Further analyses of KRT81

**Figure 3:**
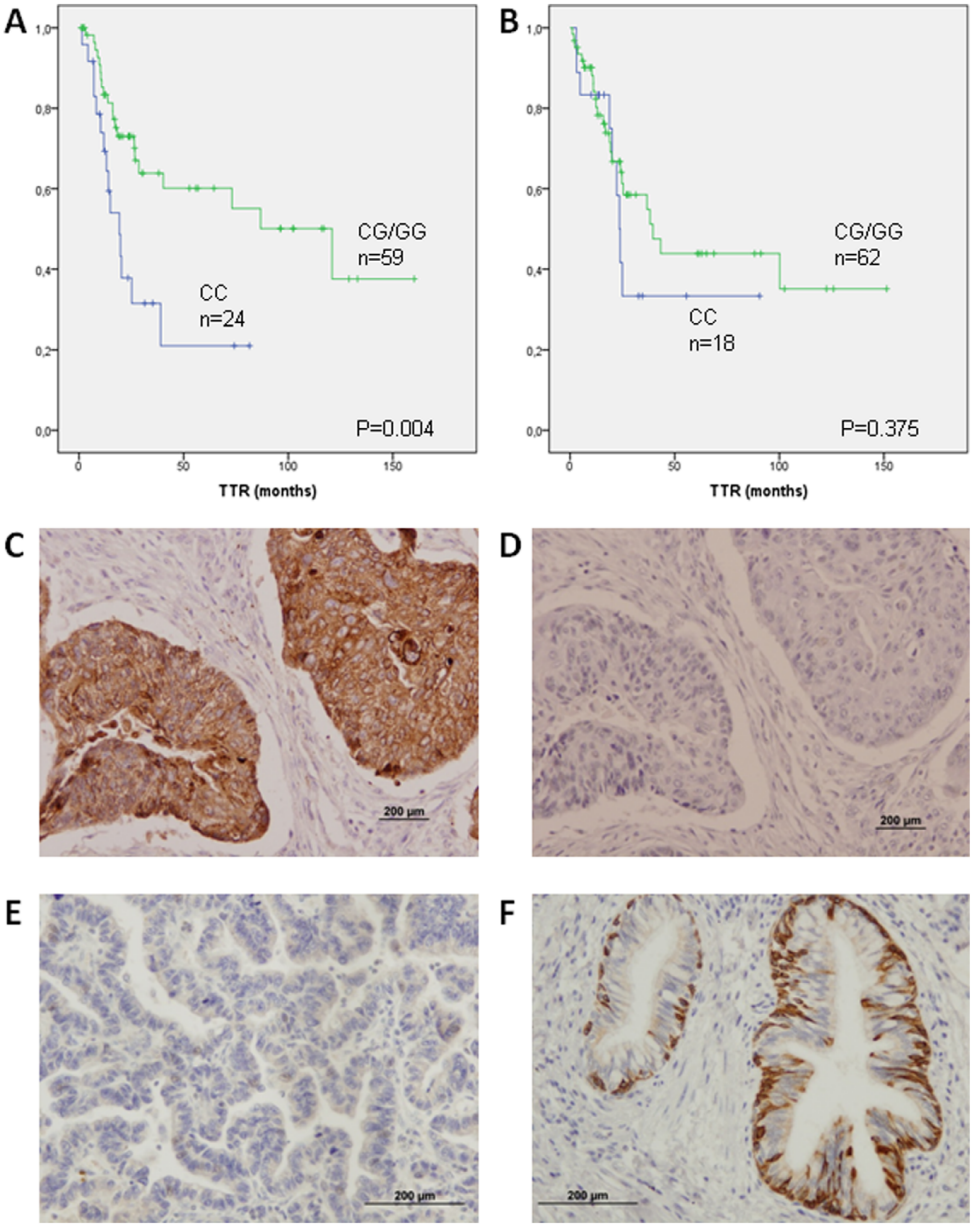
TTR according to *KRT81* rs3660 genotype in the main histological subgroups and Immunohistochemistry analysis of KRT81 protein. A: TTR according to *KRT81* rs3660 genotype in 83 of 84 squamous cell carcinoma patients; one patient could not be genotyped. B: TTR according to *KRT81* rs3660 genotype in 80 adenocarcinoma patients. C: Squamous cell carcinoma case showing diffuse cytoplasmic KRT81 staining. D: Negative control. E: Negative staining for KRT81 in an adenocarcinoma case. F: Immunohistochemistry in a normal lung tissue section. KRT81 cytoplasmic positivity in the bronchiolar epithelium was used as positive control.

Since significant differences in TTR were found according to *KRT81* rs3660 genotype, we further examined the effect of this genotype on the sub-groups of patients with adenocarcinoma and squamous cell carcinoma. Among the 83 patients with squamous cell carcinoma, TTR was 19.3 months for 24 patients with the CC genotype and 121 months for 59 patients with the CG or GG genotype (P = 0.004; Figure 3A). In contrast, no significant difference was observed according to the *KRT81* rs3660 genotype among the 80 patients with adenocarcinoma (P = 0.375; Figure 3B). We then explored the possibility of a similar differential effect for the other ten genotypes but found no differences in TTR between squamous cell carcinoma and adenocarcinoma according to genotype .

In order to further explore this marked prognostic value of the *KRT81* rs3660 genotype in squamous cell carcinoma, we analyzed KRT81 expression by immunohistochemistry in 42 squamous cell carcinoma, 33 adenocarcinoma and 2 adenosquamous carcinoma samples and in three normal lung tissue samples. Table S1 shows the results of each of the 80 samples. Notable differences were observed in the immunostaining pattern according to histological sub-type: 38 of 40 squamous cell carcinomas (95%) were positive, compared to 6 of 32 adenocarcinomas (19%) (Fisher's exact test P<0.0001; Table 5). Moreover, 3 of the 6 positive adenocarcinomas showed only a focal positivity (score 1). Sensitivity, specificity, positive predictive value, negative predictive value and accuracy were 0.95, 0.81, 0.86, 0.93 and 0.89, respectively. Figure 3C-F shows examples of the immunohistochemical evaluation in squamous cell carcinoma, adenocarcinoma and controls.

**Table 5:** Immunostaining of KRT81in squamous cell carcinomas and adenocarcinomas.

	SCC(n = 40*)	ADC(n = 32*)
**Negative (0)**	2 (5%)	26 (81%)
**Positive (1+2)**	38 (95%)	6 (19%)
**Fisher's exact test**	P<0.0001	

SCC: squamous cell carcinoma; ADC: adenocarcinoma.

*Immunostaining was not interpretable in 2 SCC and 1 ADC.

## Discussion

In the present study, we have analyzed 11 miR-SNPs in a series of 175 resected NSCLC patients and correlated our results with TTR and OS. We found that patients with the *KRT81* rs3660 CC genotype had a shorter TTR than those with the CG or GG genotype and that patients with the *XPO5* rs11077 AA genotype had a shorter TTR than those with the AC or CC genotype. These results also held true in the subgroup of patients with stage I disease. Furthermore, the multivariate analyses showed that the *KRT81* rs3660 CC genotype and the *XPO5* rs11077 AA genotype were independent prognostic variables for TTR. The univariate analysis for OS showed an association between survival and the *MIR423* rs6505162 genotype; however, this was not an independent variable in the multivariate analysis. Furthermore, the prognostic value of the *KRT81* rs3660 genotype was more marked in squamous cell carcinoma, indicating that KRT81 may be a novel immunohistochemical marker for squamous cell carcinoma of the lung.

Several miR-SNPs are known to affect lung cancer susceptibility and survival. A SNP in the pre-miR-196a2 rs11614913 was first associated with survival in NSCLC patients [23] and was later linked to a higher risk of developing lung cancer in Chinese [27] and Korean [28] populations. A SNP in the pre-miRNA flanking region *miR-30c-1* rs928508 has been related to survival in NSCLC, with a greater effect in stage I and II disease[29]. A miRNA target site SNP in the *KRAS* gene was associated with a higher risk of developing NSCLC among moderate smokers [22]. Recently, a SNP haplotype in the miRNA biogenesis gene *RNASEN* was associated with shorter survival in lung cancer [30], and a SNP in another biogenesis-related compound, *AGO1* rs636832, was linked to a decreased risk of lung cancer [31]. To date, however, no relation between miR-SNPs and lung cancer recurrence after surgery has been reported.

XPO5 is the RAN-GTP-dependent protein that transports the pre-miRNA from the nucleus to the cytoplasm. Recently, no relation was found between *XPO5* rs11077 and lung cancer risk in a Korean population[31]. In contrast, in the present study, we have observed a positive association between this SNP and TTR in a European population. We speculate that a SNP in *XPO5* could alter the normal function of the protein to extrude the pre-miRNAs from the nucleus, thus altering the regulation of multiple miRNAs in the cell.

Stage II and III NSCLC patients are routinely treated with adjuvant chemotherapy after surgical resection, which has improved survival in several randomized clinical trials [3], [32], [33], [34]. However, there are many high-risk stage I patients that could also benefit from this treatment, and it has been suggested that patients with tumors ≥4 cm can derive survival benefit from adjuvant chemotherapy [35]. Biomarkers to accurately identify stage I patients with a high risk of recurrence would be a useful tool in the management of these patients. Since the effect on recurrence observed with the genetic variants in *KRT81* and *XPO5* was maintained in the subgroup of patients with stage I disease, we suggest that these SNPs are ideal candidates for further investigation with a view to individualizing treatment in these patients.

Importantly, tumor recurrence was influenced by the SNP located in the 3′UTR of *KRT81*, the binding site of several miRNAs: miR-17, miR-93, miR-20b, miR-519d, miR-520g, miR-520h, miR-519c-3p, miR-519b-3p, miR-519a and miR-765. Some of these miRNAs have previously been shown to be altered in NSCLC [11], and the presence of the SNP affects the seed-sequence binding site of these miRNAs, as shown in Figure S4.The allelic frequency of this SNP is different in human cancers than in the normal population [36]. *KRT81* encodes for KRT81 protein, also known as Hb-1, a type of hair keratin that is physiologically expressed in hair shafts. Keratins are proteins expressed in all types of epithelial cells [37], with different expression patterns among different carcinomas [38], and they are extensively used as diagnostic markers. They constitute the intermediate filaments of epithelial cells, involved in the maintenance of cell integrity and resistance to mechanical and non-mechanical stressors [39]. Keratins are not only related to the regulation of cellular functions like motility and growth but also to protein synthesis, apico-basal polarization and intracellular signaling, and they have been described as prognostic markers in epithelial tumors [40]. Breast carcinomas ectopically express KRT81[41], and in the present study we have observed KRT81 expression in lung tumor tissue by immunohistochemistry. To date, *KRT81* has not been validated as a prognostic marker, and the present study is the first to link a *KRT81* variant to tumor recurrence.

In addition, we have found that KRT81 may well be a new immunohistochemical marker of squamous cell carcinoma. KRT81 showed a clear positive staining in squamous cell carcinomas (95% positive), whereas 81% of adenocarcinomas were negative. Interestingly, in the sub-group analyses of TTR according to histological sub-type, the effect of the *KRT81* rs3660 genotype on recurrence was more pronounced in patients with squamous cell carcinoma. The fact that several new targeted agents are activespecifically in adenocarcinoma and others should not be used in squamous cell carcinoma because of potential complications underscores the need to further classify NSCLC as squamous or non-squamous carcinoma. In this setting, KRT81 could be a useful novel marker for the differential diagnosis of NSCLC; as such, it could be added to the panel of immunohistochemical markers currently used, such as TTF1 and P63 [42], [43].

The growing evidence of links between miRNAs and cancer makes the analysis of SNPs in miRNA-related genes a potential key technique for selecting patients for risk stratification. Despite certain limitations – including the lack of independent or functional validation and the relatively limited number of SNPs – the present study is the first to observe miR-SNP-related differential patterns of tumor recurrence in surgically treated NSCLC patients. Our findings indicate that SNPs in *KRT81* and *XPO5* could prove to be useful biomarkers for individualizing therapy in NSCLC patients and that KRT81 may be a novel immunohistochemical marker of squamous cell carcinoma, providing a new diagnostic tool to be used in therapeutic decision-making.

## Supporting Information

Figure S1 TTR according to *KRT81* rs3660 genotype.
(TIF)

Figure S2 TTR according to *XPO5* rs11077 genotype.
(TIF)

Figure S3 OS according to *MIR423* rs6505162 genotype.
(TIF)

Figure S4 Predicted conserved miRNAs targeting KRT81. SNP rs3660, located in the 3′UTR region of *KRT81*, affects the binding of these miRNAs.
(PPTX)

Table S1 Immunostaining of KRT81 in the 80 cases analyzed.
(DOC)

## References

[1] Jemal A, Siegel R, Xu J, Ward E (2010). Cancer Statistics, 2010.. CA Cancer J Clin.

[2] Brundage MD (2002). Prognostic Factors in Non-small Cell Lung Cancer* : A Decade of Progress.. Chest.

[3] Arriagada R, Bergman B, Dunant A, Le Chevalier T, Pignon JP (2004). Cisplatin-Based Adjuvant Chemotherapy in Patients with Completely Resected Non–Small-Cell Lung Cancer.. N Engl J Med.

[4] Bartel DP (2004). MicroRNAs: Genomics, Biogenesis, Mechanism, and Function.. Cell.

[5] Lee YS, Dutta A (2009). MicroRNAs in Cancer.. Annu Rev Pathol.

[6] Takamizawa J, Konishi H, Yanagisawa K, Tomida S, Osada H (2004). Reduced Expression of the let-7 MicroRNAs in Human Lung Cancers in Association with Shortened Postoperative Survival.. Cancer Res.

[7] Yanaihara N, Caplen N, Bowman E, Seike M, Kumamoto K (2006). Unique microRNA molecular profiles in lung cancer diagnosis and prognosis.. Cancer Cell.

[8] Gallardo E, Navarro A, Vinolas N, Marrades RM, Diaz T (2009). miR-34a as a prognostic marker of relapse in surgically resected non-small-cell lung cancer.. Carcinogenesis.

[9] Raponi M, Dossey L, Jatkoe T, Wu X, Chen G (2009). MicroRNA Classifiers for Predicting Prognosis of Squamous Cell Lung Cancer.. Cancer Res.

[10] Hu Z, Chen X, Zhao Y, Tian T, Jin G (2010). Serum MicroRNA Signatures Identified in a Genome-Wide Serum MicroRNA Expression Profiling Predict Survival of Non-Small-Cell Lung Cancer.. J Clin Oncol.

[11] Patnaik SK, Kannisto E, Knudsen S, Yendamuri S (2010). Evaluation of MicroRNA Expression Profiles That May Predict Recurrence of Localized Stage I Non-Small Cell Lung Cancer after Surgical Resection.. Cancer Res.

[12] Landi MT, Zhao Y, Rotunno M, Koshiol J, Liu H (2010). MicroRNA Expression Differentiates Histology and Predicts Survival of Lung Cancer.. Clin Cancer Res.

[13] Duncavage E, Goodgame B, Sezhiyan A, Govindan R, Pfeifer J (2010). Use of microRNA expression levels to predict outcomes in resected stage I non-small cell lung cancer.. J Thorac Oncol.

[14] Iorio MV, Croce CM (2009). MicroRNAs in Cancer: Small Molecules With a Huge Impact.. J Clin Oncol.

[15] Esquela-Kerscher A, Slack FJ (2006). Oncomirs — microRNAs with a role in cancer.. Nat Rev Cancer.

[16] Deng S, Calin GA, Croce CM, Coukos G, Zhang L (2008). Mechanisms of microRNA deregulation in human cancer.. Cell Cycle.

[17] Mishra PJ, Mishra PJ, Banerjee D, Bertino JR (2008). MiRSNPs or MiR-polymorphisms, new players in microRNA mediated regulation of the cell: Introducing microRNA pharmacogenomics.. Cell Cycle.

[18] Mishra PJ, Humeniuk R, Longo-Sorbello GSA, Banerjee D, Bertino JR (2007). A miR-24 microRNA binding-site polymorphism in dihydrofolate reductase gene leads to methotrexate resistance.. Proc Natl Acad Sci U S A.

[19] Wu M, Jolicoeur N, Li Z, Zhang L, Fortin Y (2008). Genetic variations of microRNAs in human cancer and their effects on the expression of miRNAs.. Carcinogenesis.

[20] Ryan BM, Robles AI, Harris CC (2010). Genetic variation in microRNA networks: the implications for cancer research.. Nat Rev Cancer.

[21] Mishra PJ, Bertino JR (2009). MicroRNA polymorphisms: the future of pharmacogenomics, molecular epidemiology and individualized medicine.. Pharmacogenomics.

[22] Chin LJ, Ratner E, Leng S, Zhai R, Nallur S (2008). A SNP in a let-7 microRNA Complementary Site in the KRAS 3′ Untranslated Region Increases Non-Small Cell Lung Cancer Risk.. Cancer Res.

[23] Hu Z, Chen J, Tian T, Zhou X, Gu H (2008). Genetic variants of miRNA sequences and non–small cell lung cancer survival.. J Clin Invest.

[24] Christensen BC, Moyer BJ, Avissar M, Ouellet LG, Plaza SL (2009). A let-7 microRNA-binding site polymorphism in the KRAS 3′ UTR is associated with reduced survival in oral cancers.. Carcinogenesis.

[25] Christensen BC, Avissar-Whiting M, Ouellet LG, Butler RA, Nelson HH (2010). Mature MicroRNA Sequence Polymorphism in MIR196A2 Is Associated with Risk and Prognosis of Head and Neck Cancer.. Clin Cancer Res.

[26] Lin J, Horikawa Y, Tamboli P, Clague J, Wood CG (2010). Genetic variations in microRNA-related genes are associated with survival and recurrence in patients with renal cell carcinoma.. Carcinogenesis.

[27] Tian T, Shu Y, Chen J, Hu Z, Xu L (2009). A Functional Genetic Variant in microRNA-196a2 Is Associated with Increased Susceptibility of Lung Cancer in Chinese.. Cancer Epidemiol Biomarkers Prev.

[28] Kim MJ, Yoo SS, Choi Y-Y, Park JY (2010). A functional polymorphism in the pre-microRNA-196a2 and the risk of lung cancer in a Korean population.. Lung Cancer.

[29] Hu Z, Shu Y, Chen Y, Chen J, Dong J (2011). Genetic Polymorphisms in the Precursor MicroRNA Flanking Region and Non-Small Cell Lung Cancer Survival.. Am J Respir Crit Care Med.

[30] Rotunno M, Zhao Y, Bergen AW, Koshiol J, Burdette L (2010). Inherited polymorphisms in the RNA-mediated interference machinery affect microRNA expression and lung cancer survival.. Br J Cancer.

[31] Kim J-S, Choi YY, Jin G, Kang H-G, Choi J-E (2010). Association of a common AGO1 variant with lung cancer risk: A two-stage case–control study.. Mol Carcinog.

[32] Kato H, Ichinose Y, Ohta M, Hata E, Tsubota N (2004). A randomized trial of adjuvant chemotherapy with uracil-tegafur for adenocarcinoma of the lung.. N Engl J Med.

[33] Winton T, Livingston R, Johnson D, Rigas J, Johnston M (2005). Vinorelbine plus cisplatin vs. observation in resected non-small-cell lung cancer.. N Engl J Med.

[34] Douillard JY, Rosell R, De Lena M, Carpagnano F, Ramlau R (2006). Adjuvant vinorelbine plus cisplatin versus observation in patients with completely resected stage IB-IIIA non-small-cell lung cancer (Adjuvant Navelbine International Trialist Association [ANITA]): a randomised controlled trial.. Lancet Oncol.

[35] Strauss GM, Herndon JE, Maddaus MA, Johnstone DW, Johnson EA (2008). Adjuvant Paclitaxel Plus Carboplatin Compared With Observation in Stage IB Non-Small-Cell Lung Cancer: CALGB 9633 With the Cancer and Leukemia Group B, Radiation Therapy Oncology Group, and North Central Cancer Treatment Group Study Groups.. J Clin Oncol.

[36] Yu Z, Li Z, Jolicoeur N, Zhang L, Fortin Y (2007). Aberrant allele frequencies of the SNPs located in microRNA target sites are potentially associated with human cancers.. Nucleic Acids Res.

[37] Moll R, Franke WW, Schiller DL, Geiger B, Krepler R (1982). The catalog of human cytokeratins: patterns of expression in normal epithelia, tumors and cultured cells.. Cell.

[38] Moll R, Divo M, Langbein L (2008). The human keratins: biology and pathology.. Histochem Cell Biol.

[39] Coulombe PA, Omary MB (2002). ‘Hard’ and ‘soft’ principles defining the structure, function and regulation of keratin intermediate filaments.. Curr Opin Cell Biol.

[40] Karantza V (2010). Keratins in health and cancer: more than mere epithelial cell markers.. Oncogene.

[41] Regnier CH, Boulay A, Asch PH, Wendling C, Chenard MP (1998). Expression of a truncated form of hHb1 hair keratin in human breast carcinomas.. Br J Cancer.

[42] Kargi A, Gurel D, Tuna B (2007). The diagnostic value of TTF-1, CK 5/6, and p63 immunostaining in classification of lung carcinomas.. Appl Immunohistochem Mol Morphol.

[43] Ring BZ, Seitz RS, Beck RA, Shasteen WJ, Soltermann A (2009). A novel five-antibody immunohistochemical test for subclassification of lung carcinoma.. Modern Pathology.

[44] Hoffman AE, Zheng T, Yi C, Leaderer D, Weidhaas J (2009). microRNA miR-196a-2 and Breast Cancer: A Genetic and Epigenetic Association Study and Functional Analysis.. Cancer Res.

[45] Peng S, Kuang Z, Sheng C, Zhang Y, Xu H (2009). Association of MicroRNA-196a-2 Gene Polymorphism with Gastric Cancer Risk in a Chinese Population.. Dig Dis Sci.

[46] Schaefer A, Jung M, Mollenkopf H-J, Wagner I, Stephan C (2009). Diagnostic and prognostic implications of microRNA profiling in prostate carcinoma.. Int J Cancer.

[47] Yang H, Dinney CP, Ye Y, Zhu Y, Grossman HB (2008). Evaluation of Genetic Variants in MicroRNA-Related Genes and Risk of Bladder Cancer.. Cancer Res.

[48] Ye Y, Wang KK, Gu J, Yang H, Lin J (2008). Genetic Variations in MicroRNA-Related Genes Are Novel Susceptibility Loci for Esophageal Cancer Risk.. Cancer Prev Res (Phila).

[49] Jazdzewski K, Murray EL, Franssila K, Jarzab B, Schoenberg DR (2008). Common SNP in pre-miR-146a decreases mature miR expression and predisposes to papillary thyroid carcinoma.. Proc Natl Acad Sci U S A.

[50] Xu T, Zhu Y, Wei QK, Yuan Y, Zhou F (2008). A functional polymorphism in the miR-146a gene is associated with the risk for hepatocellular carcinoma.. Carcinogenesis.

[51] Xu B, Feng N-H, Li P-C, Tao J, Wu D (2010). A functional polymorphism inPre-miR-146agene is associated with prostate cancer risk and mature miR-146a expression in vivo.. Prostate.

