# Supplementary figures and images for: A Dual Role for KRT81: A miR-SNP Associated with Recurrence in Non-Small-Cell Lung Cancer and a Novel Marker of Squamous Cell Lung Carcinoma
Source: PLoS One. 2011 Jul 25;6(7):e22509. doi: 10.1371/journal.pone.0022509 (PMC3143163; doi:10.1371/journal.pone.0022509)

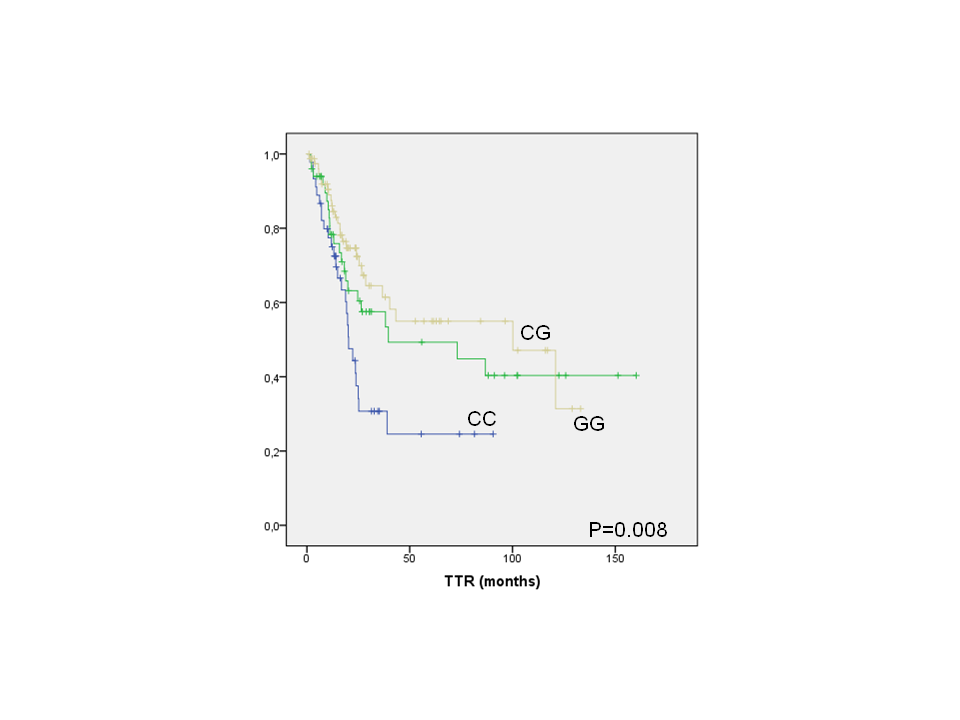

Supplement: Figure S1 — TTR according to KRT81 rs3660 genotype. (TIF) [file pone.0022509.s001.tif]

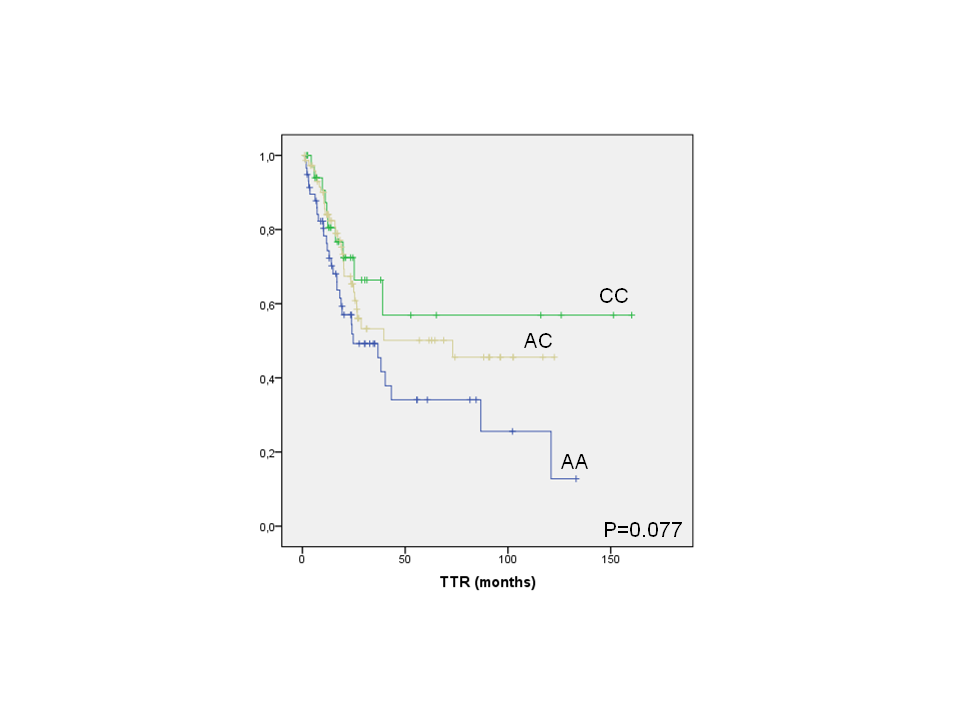

Supplement: Figure S2 — TTR according to XPO5 rs11077 genotype. (TIF) [file pone.0022509.s002.tif]

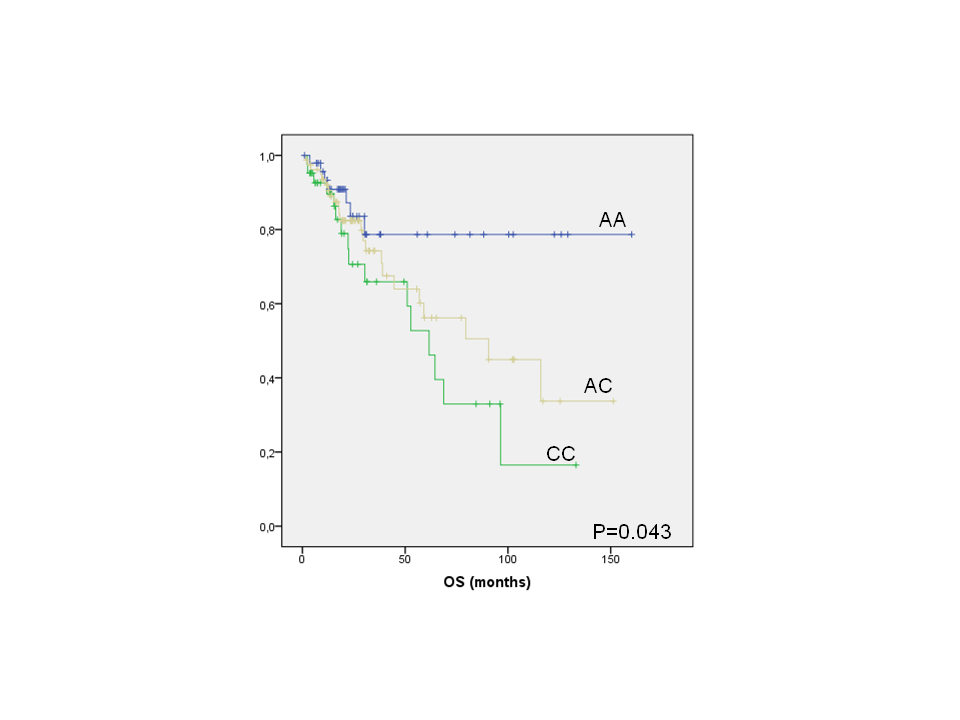

Supplement: Figure S3 — OS according to MIR423 rs6505162 genotype. (TIF) [file pone.0022509.s003.tif]
