# Supplementary material for: A Dual Role for KRT81: A miR-SNP Associated with Recurrence in Non-Small-Cell Lung Cancer and a Novel Marker of Squamous Cell Lung Carcinoma
Source: PLoS One. 2011 Jul 25;6(7):e22509. doi: 10.1371/journal.pone.0022509 (PMC3143163; doi:10.1371/journal.pone.0022509)
Supplement: Table S1 — Immunostaining of KRT81 in the 80 cases analyzed. (DOC) [file pone.0022509.s005.doc]

**Supplementary Table 1.** Immunostaining of KRT81 in the 80 cases analyzed.

| **CASE** | **HISTOLOGY** | **SCORE** | **COMMENT** |
| --- | --- | --- | --- |
| 1 | SCC | 1 |  |
| 2 | SCC | 2 |  |
| 3 | SCC | 2 |  |
| 4 | SCC | 2 |  |
| 5 | ADC | 0 |  |
| 6 | ADC | 0 |  |
| 7 | SCC | 2 |  |
| 8 | ADC | 0 |  |
| 9 | ADC | 1 |  |
| 10 | ADC | 0 |  |
| 11 | ADC | 0 |  |
| 12 | ADC | 0 |  |
| 13 | ADC | 2 |  |
| 14 | SCC | 2 |  |
| 15 | SCC | 2 |  |
| 16 | SCC | 2 |  |
| 17 | SCC | 2 |  |
| 18 | ADC | 0 |  |
| 19 | SCC | 2 | Previous neoadjuvant treatment. |
| 20 | SCC | 2 |  |
| 21 | SCC | 2 |  |
| 22 | ADC | 0 |  |
| 23 | ADC | 0 |  |
| 24 | ADC | 0 |  |
| 25 | SCC | 2 |  |
| 26 | SCC | 2 |  |
| 27 | SCC | 2 |  |
| 28 | SCC | 2 |  |
| 29 | SCC | 0 | Poorly differentiated SCC. Extensive necrotic component. |
| 30 | SCC | 2 |  |
| 31 | Adenosquamous | 0 |  |
| 32 | ADC | 0 |  |
| 33 | ADC | 0 |  |
| 34 | SCC | 2 |  |
| 35 | SCC | 2 |  |
| 36 | SCC | 2 |  |
| 37 | SCC | 0 |  |
| 38 | SCC | 2 |  |
| 39 | SCC | 2 |  |
| 40 | SCC | 2 |  |
| 41 | SCC | 2 |  |
| 42 | SCC | 2 |  |
| 43 | SCC | 2 |  |
| 44 | SCC | N/A | Extensive necrotic component. |
| 45 | SCC | 1 |  |
| 46 | SCC | 2 |  |
| 47 | Adenosquamous | 2 | Negative glandular areas and positive squamous areas. |
| 48 | SCC | 2 |  |
| 49 | SCC | 2 |  |
| 50 | SCC | N/A | Previous neoadjuvant treatment. No viable tumor. |
| 51 | SCC | 2 |  |
| 52 | SCC | 1 |  |
| 53 | SCC | 2 |  |
| 54 | SCC | 2 |  |
| 55 | ADC | N/A | Previous neoadjuvant treatment. No viable tumor. |
| 56 | ADC | 0 |  |
| 57 | ADC | 0 |  |
| 58 | ADC | 0 |  |
| 59 | ADC | 0 |  |
| 60 | ADC | 0 |  |
| 61 | ADC | 0 |  |
| 62 | ADC | 0 |  |
| 63 | ADC | 0 |  |
| 64 | ADC | 1 |  |
| 65 | ADC | 2 | Poorly differentiated adc. |
| 66 | ADC | 0 |  |
| 67 | ADC | 2 | Poorly differentiated adc. |
| 68 | ADC | 1 | Extensive degenerative areas. |
| 69 | ADC | 0 |  |
| 70 | ADC | 0 |  |
| 71 | ADC | 0 |  |
| 72 | ADC | 0 |  |
| 73 | ADC | 0 |  |
| 74 | SCC | 1 |  |
| 75 | SCC | 2 |  |
| 76 | SCC | 2 |  |
| 77 | SCC | 2 |  |
| 78 | Normal Control | - |  |
| 79 | Normal Control | - |  |
| 80 | Normal Control | - |  |

ADC: Adenocarcinoma; SCC: squamous cell carcinoma; N/A: uninterpretable immunostaining
